# Mastitis and Mammary Abscess Management Audit (MAMMA): A Survey of Patients’ Perspective on the Management of Mammary Abscesses in the UK

**DOI:** 10.1155/tbj/5901980

**Published:** 2026-02-17

**Authors:** Ronak Patel, Alona Courtney, Natasha Elysha Jiwa, Nur Amalina Che Bakri, Sophie Paterson, Daniel Richard Leff

**Affiliations:** ^1^ Department of Surgery and Cancer, Imperial College London, London, UK, imperial.ac.uk; ^2^ Department of Targeted Intervention, Division of Surgery and Interventional Sciences, University College London, London, UK, ucl.ac.uk; ^3^ HCA Healthcare UK, London, UK; ^4^ King George Hospital, Ilford, UK; ^5^ Imperial College Healthcare NHS Trust, London, UK, nhs.uk; ^6^ Patient Representative, London, UK

## Abstract

**Background:**

The recent Mastitis and Mammary Abscess Management Audit demonstrated widespread variation in the management of breast abscesses across the United Kingdom (UK), with up to one‐fifth undergoing surgical drainage rather than image‐guided needle aspiration. The impact of these practices on patient’s perspective and quality of life is unclear. This study aimed to assess patients’ experiences following breast abscess treatment, focusing on treatment modality, cosmesis, breastfeeding and quality of life.

**Methods:**

A cross‐sectional online survey was conducted between February and August 2024, aimed at UK‐wide adult women with a history of breast abscess. Descriptive and thematic analyses were performed using SPSS and NVivo software, with multiple imputation for missing data.

**Results:**

Of 172 participants, half underwent needle aspiration (50.58%), while 23.84% received surgical incision and drainage. Among those undergoing surgery, 68.29% reported prolonged wound healing, 85.37% experienced permanent scarring and a significant negative impact on their breast appearance (*p* = 0.029). Breastfeeding was disrupted in 58.12%, and 40.17% were unable to resume breastfeeding following treatment. Amongst participants who underwent surgery, 36.5% reported negative impacts on sexual well‐being, 31.7% on mental health, and 24.4% on self‐confidence. Thematic analysis revealed two major themes: repercussions of the treatment and issues with provision of care, highlighting delays in diagnosis, inadequate breastfeeding support, and negative cosmetic outcomes.

**Discussion:**

This study is the first to investigate patients’ experiences of breast abscess management, highlighting significant variability in practice and the association of worse cosmetic and breastfeeding outcomes with surgical treatment. Standardisation of care and improved patient counselling may improve patient experience and outcomes.

## 1. Introduction

A breast abscess is a common breast condition affecting up to 30% of women [1] with a significant burden on NHS resources [2]. The recent “Mastitis and Mammary Abscess Management Audit” (MAMMA) in the United Kingdom (UK) and Ireland has demonstrated wide variation in the management of breast abscesses [3]. Whilst ultrasound‐guided needle aspiration is the mainstay of care, certain UK breast units utilise high rates of incision and drainage. Indications for incision and drainage are not standardised, but are generally thought to include treatment failure of needle aspiration, multiloculation, skin necrosis and large abscesses over 5 cm^4^.

The impact of variation in practice in abscess treatment is unclear. Both surgical incision and drainage and ultrasound‐guided aspiration have consequences for patients [4–7] in terms of the length of time taken for resolution of symptoms, cosmetic outcome and impact on breastfeeding. However, the impact of different treatment approaches on patient experiences and impact on the quality of life has not previously been investigated. Improving our understanding of the sequelae of treatments from patients surveys may help towards improving uniformity in care and avoiding variation in practice across the UK.

This paper aimed to survey patients to elucidate their views and opinions regarding their experiences of breast abscess treatment including treatment modality, length of hospital treatment, impact on cosmesis, quality of life, mental health, sexual and physical well‐being, self‐confidence, breastfeeding and time away from their newborn baby. Parts of this work were presented previously in abstract form at the Association of Breast Surgery (ABS) Conference 2025 [8]. The present manuscript is the first full‐length publication of the study and includes the complete methodology, full results, and extended analyses and discussion beyond the conference abstract.

## 2. Methods

This study was reported according to the Consensus‐Based Checklist for Reporting of Survey Studies (CROSS) [9].

### 2.1. Study Design and Data Collection Methods

A cross‐sectional survey was created, consisting of 7 sections and 35 questions. The following sections were administered to all participants: demographics (Q1‐4, Q6‐7, Q13), breast abscess history (Q5, Q8‐10, Q12), breastfeeding (Q14 (screening) and if applicable Q15‐18, Q25, Q32‐33), surgical history (Q11 (screening) and if applicable Q19‐23), impact on the quality of life (Q24, Q26‐31). The target population was adult female patients with a history of breast abscess.

The survey was reviewed by the patient representative (SP) for relevance and comprehensibility of items. It was pretested online by three authors (RP, AC and NJ) to ensure comprehensibility and appropriate question flow, prior to releasing it to the public. The final version of the survey is available in the supporting information.

### 2.2. Sample Characteristics

The study was aimed at a UK‐wide population. Inclusion criteria comprised:

1. Adult female patients (18 years and older)
2. Previous history of a breast abscess.

Exclusion criteria were:

1. Children under 18 years of age
2. Patients with learning disabilities, severe neuropsychiatric illness or dementia
3. Patients who were unconscious, very severely ill or with terminal illness
4. Not fluent in English.

Convenience sampling technique was used to recruit volunteer participants. A target sample size of 200 participants was based on previous similar study designs in breast surgery [10].

### 2.3. Survey Administration

The survey was administered online using Qualtrics software (Version: August 2024) [11] between the 28^th^ of February and the 31^st^ of August 2024. The prevention of the multiple submission function was enabled to obviate participants from taking the survey on multiple occasions. The bot detection function was enabled to prevent bots from taking the survey.

The survey link was distributed via personal Instagram pages with high following rates (SP and ACB) and via Facebook maternity groups. These social media channels were chosen to ensure sampling within appropriate user demographics to cover the target population. The MAMMA study demonstrated that the median age of women with lactational mastitis and breast abscess was 33 (IQR 29–35) years old, and nonlactational disease was 40 (IQR 32–51) years old [3]. Of Instagram users, 32% are 18–29 years old, 32% are 30–39 years old and 20% are 40–49 years old [12]. Of Facebook users, 20% are 18–29 years old, 29% are 30–39 years old and 22% are 40–49 years old [12].

### 2.4. Study Preparation and Ethical Considerations

The study advertising material was designed and reviewed by the authors (AC, NJ and ACB), the patient representative (SP) and the ethical committee to ensure clear call to action. Ethical approval was obtained from Imperial College Research Ethics Committee (ICREC) (Application ID 6920555). All surveys were anonymous. Informed consent was implied by participants upon proceeding with the survey. No personal identifiable information was collected. Only the research group had access to the survey data.

### 2.5. Statistical Analysis

SPSS [13] was used to perform statistical analysis. Frequencies of observation were summarised using descriptive statistics. Categorical data were compared using the chi‐squared test. A *p* value of < 0.05 was considered statistically significant. NVivo14 [14] was used for qualitative data analysis.

Branching logic was used within the survey to ensure that relevant questions were asked of specific cohorts: (1) impact on breastfeeding for women who breastfed at the time of the abscess and (2) details of the surgical treatment for those selecting incision and drainage. The rate of missing data was determined in SPSS [13] for each survey question within these cohorts.

### 2.6. Database Cleaning

Prior to commencing data analysis, the survey data was cleaned. Records considered to be meaningless were removed, including (a) no data, (b) only demographic data, (c) survey completion time of less than 60 s or (d) if more than 50% of the data were missing. Participants who had no history of breast abscess were removed from the analysis as they did not meet the inclusion criteria.

### 2.7. Data Missingness Analysis

Prior to analysing “missingness”, data were split into four cohorts to account for conditional question branching: (a) participants who breastfed and had a surgical treatment (Cohort 1), (b) participants who breastfed and had no surgical treatment (Cohort 2), (c) participants who did not breastfeed and had a surgical treatment (Cohort 3), and (d) participants who did not breastfeed and had no surgical treatment (Cohort 4). Where < 5% of data were missing, the multiple imputation method was used [15]. Where 5%–40% of data were missing, item nonresponse error was assessed using Little’s test of missingness to determine the significance of missing values. If the outcome of Little’s test of missingness was a *p* value of > 0.05, then the null hypothesis was accepted, and the missing data were assumed to be missing completely at random (MCAR). MCAR missing data were replaced using multiple imputation to avoid the bias associated with using the deletion method [15]. If Little’s test of missingness suggested significance, then data were assumed to be missing at random (MAR), since it is impossible to differentiate it from missing not at random (MNAR) [16], and multiple imputation was performed. Complete cases were compared to imputed cases using the chi‐square test. Weighting of items was not used during the analysis of the data. Unit nonresponse error could not be directly assessed because of the type of distribution channel selected.

### 2.8. Thematic Analysis

Free text questions addressed the following areas: (1) the effect of surgery on the appearance of breasts, (2) postoperative scarring and (3) negative repercussions of treatment on life (Questions 22, 23 and 24, supporting information, survey questions). Free text data were extracted and imported into NVivo14 [14]. Thematic analysis was performed according to Braun and Clarke methodology [17]. The process of familiarisation with the data was followed by the initial coding of the entire dataset, performed by one researcher (AC). Subsequently, codes were collated into themes. These were reviewed, re‐named, defined and discussed with the study team (AC, RP and DRL). Illustrative example quotes were provided for each theme.

## 3. Results

### 3.1. Demographics

The survey was viewed and/or attempted by 286 unique participants, of which 55 (19.23%) records had no data, 8 (2.80%) records contained only demographic data, 3 (1.05%) records were completed in less than 60 s, and 7 (2.45%) records had more than 50% of data missing. Participants, who reported no previous history of a breast abscess (*n* = 41, 14.34%), were excluded from the analysis because they did not meet the study inclusion criteria. In total, 172 (60.14%) survey records were included in the analysis. A third of these women reported having a recurrent breast abscess (*n* = 56, 32.56%). Demographics of participating women can be found in Table 1. Briefly, 157 (91.28%) women were aged between 25 and 55 years, 95 (55.23%) women were white Caucasian and 65 (37.79%) women were Asian; 160 (93%) women were married and 87 (50.58%) women were in full‐time employment. Detailed data missingness analysis can be found in the Supporting Information (available here).

**Table 1:** Demographics and treatment.

	History of breast abscess *n* = 172 (%)	I&D *n* = 41 (%)	Needle aspiration *n* = 87 (%)
*Age*			
18–24	1 (0.6)	1 (2.4)	0
25–34	51 (29.7)	14 (34.1)	27 (31.0)
35–44	73 (42.4)	17 (41.5)	41 (47.1)
45–54	33 (19.2)	7 (17.1)	12 (13.8)
55–64	8 (4.7)	1 (2.4)	4 (4.6)
65 or over	6 (3.5)	1 (2.4)	3 (3.4)

*Ethnicity*			
White (British)	63 (36.6)	14 (34.1)	40 (46.0)
White (other)	32 (18.6)	9 (22.0)	16 (18.4)
Black/African/Caribbean	2 (1.2)	1 (2.4)	0
Asian	65 (37.8)	16 (39.0)	27 (31.0)
Mixed/Multiple ethnic groups	3 (1.7)	1 (2.4)	0
Other	5 (2.9)	0	3 (3.4)
Chinese	2 (1.2)	0	1 (1.1)

*Relationship status*			
Married	160 (93.0)	38 (92.7)	84 (96.6)
Widowed	1 (0.6)	0	0
Divorced	3 (1.7)	2 (4.9)	1 (1.1)
Separated	3 (1.7)	0	1 (1.1)
Single	5 (2.9)	1 (2.4)	1 (1.1)

*Employment status*			
Full‐time employment	87 (50.6)	24 (58.5)	45 (51.7)
Part‐time employment	26 (15.1)	5 (12.2)	14 (16.1)
Unemployed	21 (12.2)	4 (9.8)	13 (14.9)
Self‐employed	24 (14.0)	6 (14.6)	8 (9.2)
Student	1 (0.6)	1 (2.4)	0
Retired	13 (7.6)	1 (2.4)	7 (8.0)

*Disability*			
Yes	5 (2.9)	2 (4.9)	0
No	166 (96.5)	39 (95.1)	87 (100)
Prefer not to say	1 (0.6)	0	0

*Hospital admission*			
No	110 (64.0)	9 (22.0)	51 (58.6)
1 night	20 (11.6)	11 (26.8)	10 (11.5)
2–3 nights	22 (12.8)	9 (22.0)	14 (16.1)
> 3 nights	20 (11.6)	12 (29.3)	12 (13.8)

*IV Antibiotics*			
One off dose	16 (9.3)	5 (12.2)	8 (9.2)
No	103 (59.9)	10 (24.4)	51 (58.6)
Not sure	18 (10.5)	10 (24.4)	8 (9.2)
Multiple doses	35 (20.3)	16 (39.0)	20 (23.0)

*Needle aspiration*			
Once	29 (16.9)	10 (24.4)	29 (33.3)
2–3 times	27 (15.7)	8 (19.5)	27 (31.0)
> 3 times	31 (18.0)	7 (17.1)	31 (35.6)
No	79 (45.9)	10 (24.4)	0
Not sure	6 (3.5)	6 (14.6)	0

*Incision and Drainage*			
Yes	41 (23.8)	41 (23.8)	25 (28.7)
No	129 (75.0)	0	60 (69.0)
Not sure	2 (1.2)	0	2 (2.3)

### 3.2. Type of Treatment

A third of women were admitted to the hospital (*n* = 62, 36.05%) and given intravenous (IV) antibiotics (*n* = 51, 29.65%). Half of respondents received needle aspiration (*n* = 87, 50.58%), of which 29 (16.86%) women underwent single aspiration, 27 (15.70%) women required 2‐3 aspirations, and 31 (18.02%) women needed more than 3 aspirations. A quarter of these women (*n* = 25, 28.74%) were unsuccessfully treated with needle aspiration and required an incision and drainage to resolve their abscess (Table 1).

### 3.3. Surgical Treatment

One‐fifth of women (*n* = 41, 23.84%) received incision and drainage (Table 2). Half of these women reported prolonged wound healing of over four weeks (*n* = 28, 68.29%), requiring at least five dressing changes (*n* = 31, 75.61%) and over five additional hospital visits (*n* = 10, 24.39%). A substantial proportion of women reported that surgical incision drainage has affected the appearance of their breast (*n* = 28, 68.29%), with the majority confirming presence of permanent scaring after the surgery (*n* = 35, 85.37%).

**Table 2:** Surgical treatment.

	**All I&D *n* = 41 (%)**	**I&D + needle aspiration *n* = 25 (%)**

*Wound healing after I&D*		
< 2 weeks	3 (7.3)	1 (4.0)
2–4 weeks	10 (24.4)	7 (28.0)
4–8 weeks	13 (31.7)	6 (24.0)
> 8 weeks	15 (36.6)	11 (44.0)

*How many dressing changes after I&D*		
< 5	10 (24.4)	6 (24.0)
5–10	17 (41.5)	9 (36.0)
> 10	14 (34.1)	10 (40.0)

*Additional hospital visits after I&D*		
1–2	13 (31.7)	9 (36.0)
2–5	8 (19.5)	6 (24.0)
> 5	10 (24.4)	6 (24.0)
0	10 (24.4)	4 (16.0)

*Has the surgery affected the appearance of your breast?*		
Yes	28 (68.3)	17 (68.0)
No	11 (26.8)	6 (24.0)
Not sure	2 (4.9)	2 (8.0)

*Has the surgery resulted in any scarring?*		
Yes	35 (85.4)	20 (80.0)
No	4 (9.8)	3 (12.0)
Not sure	2 (4.9)	2 (8.0)

### 3.4. Impact on Breastfeeding

Over half of the respondents with a breast abscess were breastfeeding at the time of the diagnosis (*n* = 117, 68.02%). Of these, only 76 (64.96%) women were given breastfeeding advice. Many women had to pause breastfeeding from an affected breast (*n* = 68, 58.12%), of whom a third were unable to restart breastfeeding after the resolution of the abscess (*n* = 47, 40.17%) or reported having a negative impact on their ability to breastfeed long‐term (*n* = 44, 37.61%). Having a breast abscess affected both their confidence (*n* = 60, 51.28%) and their desire (*n* = 50, 42.74%) to breastfeed in the future. Many women reported that treatment led to separation from their newborn baby (*n* = 42, 35.90%), typically because they were admitted to the hospital and/or required surgery. Just under a quarter of women (*n* = 27, 23.08%) felt that having a breast abscess had negatively affected their ability to bond with their baby (Table 3).

**Table 3:** Impact on breastfeeding (for those breastfeeding only).

	**Breastfeeding at the time of breast abscess *n* = 117 (%)**	**I&D *n* = 30 (%)**	**Needle aspiration *n* = 64 (%)**

*Did you have to pause breast-feeding from the affected breast?*			
Yes, did not restart	47 (40.2)	20 (66.7)	30 (46.9)
Yes, able to restart	21 (17.9)	2 (6.7)	8 (12.5)
No, carried on	48 (41.0)	7 (23.3)	26 (40.6)
Not sure	1 (0.9)	1 (3.3)	0

*Has it impacted your ability to breastfeed long-term?*			
Yes	44 (37.6)	15 (50.0)	26 (40.6)
No	57 (48.7)	11 (36.7)	29 (45.3)
Not sure	16 (13.7)	4 (13.3)	9 (14.1)

*Has it affected your confidence to breastfeed in the future?*			
Yes	44 (37.6)	17 (56.7)	27 (42.2)
Maybe	16 (13.7)	6 (20.0)	9 (14.1)
No	50 (42.7)	6 (20.0)	22 (34.4)
Not sure	7 (6.0)	1 (3.3)	6 (9.4)

*Has it affected your desire to breastfeed in the future?*			
Yes	35 (29.9)	11 (36.7)	23 (35.9)
Maybe	15 (12.8)	1 (3.3)	9 (14.1)
No	61 (52.1)	15 (50.0)	28 (43.8)
Not sure	6 (5.1)	3 (10.0)	4 (6.3)

*Did you have to separate from your baby to undergo treatment?*			
Yes, admitted to hospital	31 (26.5)	16 (53.3)	21 (32.8)
Yes, underwent surgery	11 (9.4)	8 (26.7)	9 (14.1)
No	73 (62.4)	6 (20.0)	34 (53.1)
Not sure	2 (1.7)	0	0

*My treatment negatively affected my ability to bond with my baby*			
Strongly disagree	25 (21.4)	6 (20.0)	14 (21.9)
Disagree	45 (38.5)	11 (36.7)	24 (37.5)
Neither agree, nor disagree	20 (17.1)	5 (16.7)	9 (14.1)
Agree	18 (15.4)	5 (16.7)	12 (18.8)
Strongly agree	9 (7.7)	3 (10.0)	5 (7.8)

### 3.5. Impact on Quality of Life

Respondents who had incision and drainage or needle aspiration reported a negative effect on mental health, sexual and physical health in about a third of responses and a negative effect on self‐confidence in over a fifth of responses (Table 4). A statistically significant difference was observed in the satisfaction with the breast appearance when patients who underwent the abscess incision and drainage were compared with those who did not require an operation (*χ* ^2^ = 17.15, *p* = 0.029).

**Table 4:** Quality of life.

	**History of breast abscess *n* = 172 (%)**	**I&D *n* = 41 (%)**	**Needle aspiration *n* = 87 (%)**

*How satisfied are you with the overall outcome of the treatment you received for your breast abscess?*			
Extremely dissatisfied	24 (14.0)	9 (22.0)	11 (12.6)
Somewhat dissatisfied	34 (19.8)	5 (12.2)	16 (18.4)
Neither satisfied nor dissatisfied	39 (22.7)	9 (22.0)	17 (19.5)
Somewhat satisfied	41 (23.8)	8 (19.5)	24 (27.6)
Extremely satisfied	34 (19.8)	10 (24.4)	19 (21.8)

*How satisfied are you with your breasts now compared to before you had your breast abscess?*			
Extremely dissatisfied	18 (10.5)	4 (9.8)	7 (8.0)
Somewhat dissatisfied	38 (22.1)	17 (41.5)	25 (28.7)
No change	68 (39.5)	10 (24.4)	30 (34.5)
Somewhat satisfied	26 (15.1)	3 (7.3)	12 (13.8)
Extremely satisfied	22 (12.8)	7 (17.1)	13 (14.9)

*My breast abscess treatment has left me negatively affected in the following areas:*			
**Mental health/well-being**			
Strongly disagree	32 (18.6)	6 (14.6)	15 (17.2)
Disagree	40 (23.3)	5 (12.2)	14 (16.1)
Neither agree nor disagree	51 (29.7)	17 (41.5)	31 (35.6)
Agree	38 (22.1)	12 (29.3)	21 (24.1)
Strongly agree	11 (6.4)	1 (2.4)	6 (6.9)

**Sexual well-being**			
Strongly disagree	43 (25.0)	8 (19.5)	18 (20.7)
Disagree	34 (19.8)	6 (14.6)	17 (19.5)
Neither agree nor disagree	46 (26.7)	12 (29.3)	22 (25.3)
Agree	41 (23.8)	14 (34.1)	25 (28.7)
Strongly agree	8 (4.7)	1 (2.4)	5 (5.7)

**Self confidence**			
Strongly disagree	35 (20.3)	4 (9.8)	14 (16.1)
Disagree	33 (19.2)	8 (19.5)	17 (19.5)
Neither agree nor disagree	64 (37.2)	19 (46.3)	35 (40.2)
Agree	27 (15.7)	10 (24.4)	14 (16.1)
Strongly agree	13 (7.6)	0	7 (8.0)

**Physical well-being**			
Strongly disagree	35 (20.3)	7 (17.1)	18 (20.7)
Disagree	37 (21.5)	9 (22.0)	16 (18.4)
Neither agree nor disagree	44 (25.6)	15 (36.6)	25 (28.7)
Agree	41 (23.8)	9 (22.0)	22 (25.3)
Strongly agree	15 (8.7)	1 (2.4)	6 (6.9)

*Has the treatment had any other negative repercussions in your life?*			
Yes	45 (26.2)	12 (29.3)	23 (26.4)
No	108 (62.8)	22 (53.7)	54 (62.1)
Not sure	19 (11.0)	7 (17.1)	10 (11.5)

### 3.6. Thematic Analysis

Free text questions were used in the survey to investigate (1) the effect of surgery on breast appearance, (2) scarring after surgery and (3) negative repercussions of treatment on life. Participants also had an opportunity to leave additional comments.

Two major themes emerged from the analysis of the free text answers: (1) repercussions of treatment and (2) issues with provision of care. Participants reported repercussions in terms of physical effects, negative impact on mental health, breastfeeding, baby, daily activities and change in breast appearance. The key physical effect was pain associated with having an abscess, its treatment and the subsequent long‐term breast pain. The degree of pain has been described as “severe”, “unbearable”, and “excruciating”. One participant described that the pain of her abscess drainage was *“more painful than anything else I have felt”.* The impact on mental health ranged from anxiety to feeling distressed, overwhelmed, dismissed, disappointed and angry. Women reported being traumatised by their treatment: *“I am scared and traumatised to have another child because I have to breastfeed*”. Another described her experience as “*really harrowing”.*

Some women were left unable to breastfeed from the affected breast, and others felt scared about breastfeeding and reported they *“would never breastfeed again”*. Women described difficulty in juggling childcare to attend hospital appointments, frequent dressing changes and the impact of taking time off work during sickness on their income: “*Time off work, affecting my income has had a huge impact”*.

The impact on the appearance of their breasts ranged from scarring to changes in volume, shape, breast sensation and nipple shape and position. Women described different types of scars: small, *“massive”*, uneven, lumpy, and keloid. Some had multiple scars. One participant said that her breast *“looked butchered”.* Women reported a change in the size of the affected breast from being *“a bit smaller than before” to “slightly larger”*. They reported “asymmetry following the surgery” and *“indentation where the cuts have been”*. For some, surgery led to *“nipple moving a bit to the side because of the scar”*, others had a “*dropped nipple”* or “*altered nipple shape”*. Participants described feeling lumps in their breasts. The treatment for breast abscess affected their body confidence and altered their choices of bras and tops: “I no longer wear bras I enjoy, just ones that are comfortable”.

Interestingly, the theme of “issues with provision of care” that participants received emerged completely unprompted, as this was not part of the questionnaire. The key issues appear to be a delay in diagnosis and treatment, inappropriate treatment and advice relating to breastfeeding. Women reported a lack of support and appropriate provision of information. Women expressed concerns about a lack of awareness of this issue amongst healthcare staff. Detailed descriptions of themes and illustrative quotes are provided in Table 5.

**Table 5:** Thematic analysis.

Major theme	Minor theme	Category	Code	Example quotes
Repercussions of the treatment	Change in breast appearance	Change in how breast feels	Abnormal skin	“The skin area is firm and not as usual as my wound was an open one.” “Very small line hardly visible with slight darkened area around”
			Breast lumps	“I’ve had lumps in my breast after the first time I had abscesses” “A hard lump has formed” “I had a lump on my first pregnancy which went away, by itself. On my second pregnancy it returned but again I thought went away. By my third pregnancy it was 6 cm in size. Very painful, and added worry to an already stressful pregnancy.”
			Breast swelling	“Constantly being unable to do things as it’s so swollen and painful”
		Change in nipple	Position	“Dropped nipple” “my nipple moved a bit to the side because of the scar”
			Shape	“Altered nipple shape” “Yes my nipple looks flat at times on my breast, like it’s pushed in”
		Change in breast shape	Asymmetry	“Breast were asymmetric following the surgery”
			Indentation	“It also looks as if there is a indent in my breast on one side” “indentation where the cuts have been”
		Change in volume	Larger volume	“reduced volume”
			Smaller volume	“breast size (a bit smaller than before)” “reduced volume”
		Discharge from breast		“My breasts leaked for almost 2 years”
		Needing subsequent corrective procedure		“Breast were asymmetric following the surgery, until corrected with an augmentation”
		Scarring	Keloid scar	“I had an over granulating wound which took 8 months to heal by secondary intention and now have a large keloid scar”
			Lumpy scar	“The scar did not heal well and left a lumpy appearance at the incision site.”
			Multiple scars	“I have two scars on my left breast from the two abscesses” “got 2 surgery in 1 week because the first one didn’t go well, so i have 2 scars”
			Size of scar	“Massive scar near my nipple” “I now have a scar just short of two inches along my areola.” “Small scar”
			Uneven scar	“Uneven scarring at incision site.”
			Visible scar	“Now have various scars on my breasts.” “Scar along the side of my nipple. Thin but visible.”
	Negative impact on baby	From anaesthetic during pregnancy		“I was pregnant at the time and extremely stressed about potential effects of the anaesthesia on the baby.”
		From inability to breastfeed		“I was left unable to breastfeed from the affected breast but continued to feed from the other breast. This led to issues with my baby not gaining enough weight as my supply from one breast was not enough.”
	Impact on breastfeeding	Negative emotions about stopping breastfeeding		“The lack of accurate diagnosis at the right time and viable options for treatment without completely stopping breastfeeding was hard to deal with”
		Preoccupation with breastfeeding		“I was pregnant with second child 2 years later ‐ I was fixed about breastfeeding, looking after my boots, to the point that I was harvesting colostrum and freezing it in the late pregnancy so I could provide the best nutrition to my second child.”
		Fear about future breastfeeding		“I am scared and traumatised to have another child because I have to breastfeed”
		Needing to stop breastfeeding		“got this when i have my first born and now my second and 3rd baby they only want to breast feed from one boobs (the non surgery boobs)” “I was left unable to breastfeed from the affected breast but continued to feed from the other breast.” “I would never breastfeed again” “now i’m only breast feed my other baby in one breast because they refused to feed in my boobs that used to be infected”
	Impact on daily activities	Choice of clothes		“Carefully select what tops I wear.” “I no longer wear bras I enjoy just ones that are comfortable”
		Frequent dressing changes		“Only treatment of my open wound was bandages applied every few days on visits to the hospital.”
		Impact on childcare to attend treatment		“Childcare for attending appointments”
		Impact on work and income		“Having to take time off of work for recovery.” “Time off work, effecting my income has had a huge impact.”
		Unpleasantness of dealing with pus		“By the time i got an appointment at the hospital i had an open wound on my breast where my body was draining it, itself. Constant puss flowing for days.”
	Negative effect on mental health	Anxiety	Being unwell	“it’s all I can think about, being admitted to hospital again without my baby or my toddler and being that unwell for such a long timeframe.”
			Recurrence	“I’m breastfeeding again with my newborn and I worry about having them again and what those lumps could do.”
			Being admitted	“I was really worried when they tried to admit me without my 4 week old baby and so I refused.” “it’s all I can think about, being admitted to hospital again without my baby or my toddler and being that unwell for such a long timeframe.”
		Body confidence		“Body confidence.”
		Feeling angry		“Still very angry about how ill I became before I was diagnosed and treated.”
		Feeling disappointed		“I received antibiotics but no opportunity to discuss my concerns with a healthcare professional. This left me feeling disappointed in the health service and with unresolved anxiety about why this often happens to me.”
		Feeling dismissed		“I was just told it was nothing and wasn’t properly treated at all.” “Midwife at home wasn’t interested in helping she told me if I didn’t continue feeding I would end up in hospital. She was right but pain was unbearable and I’d had a difficult birth. I never saw that midwife again she never came back to see me at home.”
		Feeling distressed		“This was very distressing.”
		Feeling overwhelmed		“Added to the first baby feelings of overwhelm”
		Feeling traumatised		“This has left me traumatised.” “my experience with breast abscesses after my first baby was really harrowing.” “I am scared and traumatised to have another child because I have to breastfeed”
	Physical effects	Adverse effect from medication		“Asked for medication to stop lactation and almost died from it” “I took antibiotics for over 2 months along with needle aspirations to clear the abscess however I have had a sensitive stomach after the antibiotics” “The morphine I was given in hospital had various side effects, I thought I was having a mental breakdown and was depressed.”
		Linking to subsequent breast cancer		“I don’t know if this is connected, but I’m now 54 and had breast cancer in the same breast. Have now had a mastectomy, and just finished adjuvant chemotherapy and radiotherapy.” “My sister had hospital treatment for an abscess with her first born at the age of 27, now at the age of 45, she’s just had a mastectomy for breast cancer and her cancer specialist linked it to her abscess as it was in the same milk duct and believes her cells changed and the cancer had been slow growing for years, she is still receiving treatment and will ultimately have breast reconstruction”
		Pain		“Was in severe pain.” “The pain of the drainage of the abscess was more painful than anything else I have felt.” “I had breast pain for 3 years after I had the abscess. Every month, some days before I have the period I felt pain in the place I had the abscess.” “pain was unbearable”
		Poor wound healing		“The scar did not heal well”
		Recurrence		“It would happen every other month and would lead to extremely high temperatures leaving me bed bound and in excruciating pain.”

Issues with provision of care	Delay in diagnosis			“It took 4 months and 4 hospital visits for doctors to diagnose me with breast a breast abscess.” “The only issue I would have is with the amount of time taken to refer me for further investigation; by the time I had a hospital appointment there was no alternative other than surgery as the abscesses were too far gone.” “It took a while for the doctor to recognise how serious it was, but once admitted the surgery was successful.”
	Inappropriate treatment			“My abscess came due to late diagnosis of mastitis and not the correct antibiotic being given” “I was routinely given courses of antibiotics and told repeatedly that I needed to feed through it before anyone diagnosed the abscess.” “They continually offered my incorrect advice (re‐feeding) and more than once tried to prescribe antibiotics that I should not have had when feeding (for babies sake).”
	Lack of information			“I don’t think we are educated enough on how to detect a blocked duct as I was far more aware with my 2nd and 3rd of how to avoid it happening again” “When I had the abscess I was not pregnant/BF and therefore very concerned about why it had happened. I received antibiotics but no opportunity to discuss my concerns with a healthcare professional.”
	Lack of awareness amongst healthcare staff			“I was absolutely shocked at the lack of knowledge surrounding breast abscesses/breastfeeding and lactating women by the local surgical and breast surgeon.” “No one seemed to know anything about it and it was hard to get diagnosed.” “There doesn’t appear to be enough awareness of it.”
	Lack of support			“Midwife at home wasn’t interested in helping”
	Inappropriate breastfeeding advice			“They continually offered my incorrect advice (re‐feeding)”

## 4. Discussion

This study sheds light on patient experiences and perspectives regarding management of breast abscesses. Our findings reveal considerable variability in practice whilst highlighting the sizeable proportion of women who undergo surgical incision and drainage which in turn appears to be associated with both negative cosmetic and negative satisfaction outcomes. Regardless of treatment modality, current practice is associated with an adverse effect on breastfeeding in particular. This has powerful implications for current and future clinical practice with regards to standardising and improving current and future practices in breast abscesses management.

The results here corroborate previous findings from MAMMA [3] with concordant rates of hospital admissions (25%) and surgical incision and drainage (22%), thus substantiating prior conclusions regarding unexpectedly high rates of surgical intervention. Critically, the results of the current survey illuminate the adverse impact on quality of life associated with surgical management. Two‐thirds of patients who underwent surgical incision and drainage reported the appearance of their breast was affected, and the majority reported permanent scarring which is in keeping with prior literature [18]. Furthermore, one‐quarter of patients required more than five follow‐up hospital visits, commensurate with literature reporting the economic burden of operative management compared to nonoperative management [19]. Therefore, unsurprisingly, surgical incision and drainage was coupled with significantly inferior satisfaction compared to that undergoing needle aspiration. This suggests the need for a standardised approach to breast abscess management, with avoidance of surgical incision and drainage where possible. Clear guidelines and formalised indications for operative intervention would provide clinicians with greater confidence to avoid surgery where appropriate and potentially improve patient experience, satisfaction and patient‐reported outcomes.

Appropriate counselling must also be considered in the wider context of present‐day breast abscess management, and the current findings suggest that existing approaches are not providing due diligence and attention to support holistic patient care. In particular, there appears to be a clear evidence of a negative impact on breast feeding, and a trend towards poor outcomes, regardless of modality, on mental health and self‐confidence. Substantial proportions of women reported interruptions to breast‐feeding regardless of treatment approach, as well as a detrimental impact on future and longer‐term breast‐feeding. This is of particular concern, considering the myriad of physiological and psychological benefits of breastfeeding to mother and baby [20–22]. Current advice on the NHS website [23] with regards to breastfeeding during treatment of a breast abscess is limited to two sentences which is evidently insufficient. Improving current care protocols could include the integration of lactational and psychological support to help offset these adverse outcomes. Healthcare services should have arrangements in place to prevent separation of mothers from their baby to further avoid damaging the mother‐baby bond. Although this is part of NICE recommendations [24], in practice it can rarely be achieved on the ground [3].

One of the main limitations of this study is the potential for recall bias given that this study did not collect data on when the participants experienced their breast abscess. Similarly, as with many qualitative studies, there is a limited depth of data. It would be challenging to fully explore more nuanced issues within the design and scope of this survey e.g., whether patients are recalling an episode of mastitis rather than a breast abscess per se. We also note the unavailability of data on 49 respondents, who did not undergo needle aspiration or incision and drainage or had IV antibiotics. Furthermore, as this study was distributed online, the respondents may not be representative of the broader population (e.g., elderly), thus limiting the generalisability of the results. Performing multiple imputation on missing data has the potential to ignore uncertainty and underestimate the variance in the results.

In conclusion, this study highlights significant variability in breast abscess management, with surgical incision and drainage linked to worse outcomes compared to needle aspiration and both treatments negatively impacting breastfeeding and mental health. This emphasises the need for standardised treatment guidelines, improving access to needle aspiration, minimising surgery, and integrating holistic support to improve patient care and outcomes.

## Funding

No funding was received for this research.

## Disclosure

A preliminary abstract describing parts of this study was presented at the Association of Breast Surgery (ABS) Conference 2025 [8]. The abstract was subsequently published online [25]. This submission substantially expands upon that abstract and reports the complete methods, analyses, and results.

## Conflicts of Interest

The authors declare no conflicts of interest.

## Supporting Information

Survey questions and data missingness analysis.

## Supporting information


**Supporting Information** Additional supporting information can be found online in the Supporting Information section.

## Data Availability

The data that support the findings of this study are available on request from the corresponding author. The data are not publicly available due to privacy or ethical restrictions.
