# Supplementary material for: Mastitis and Mammary Abscess Management Audit (MAMMA): A Survey of Patients’ Perspective on the Management of Mammary Abscesses in the UK
Source: Breast J. 2026 Feb 17;2026:5901980. doi: 10.1155/tbj/5901980 (PMC12910386; doi:10.1155/tbj/5901980)
Supplement: Supplementary file 1 — Supporting Information Additional supporting information can be found online in the Supporting Information section. [file TBJ-2026-5901980-s001.zip › supplementary-material .docx]

**Title**: Mastitis and Mammary Abscess Management Audit (MAMMA): a survey of patients’ perspective on the management of mammary abscesses in the UK.

**Authors**:

Ronak Patel MBChB BSc (Hons) PhD FRCS^1,a^, Alona Courtney MBChB BSc (Hons) MSc MRCS^2,3,a^, Natasha Elysha Jiwa MBBS BSc (Hons) PhD FRCS^4^, Nur Amalina Che Bakri MBChB BMedSc(Hons) MPhil^1,5^, Sophie Paterson^6^, Daniel Richard Leff MBBS FRCS MS PhD ^1,5^

1. Department of Surgery & Cancer, Imperial College London, London, United Kingdom
2. Department of Targeted Intervention, Division of Surgery & Interventional Sciences, University College London, London, United Kingdom
3. HCA Healthcare UK, London, United Kingdom
4. King George Hospital, Ilford, United Kingdom
5. Imperial College Healthcare NHS Trust, London, United Kingdom
6. Patient Representative, London, United Kingdom

a. Joint first authors - contributed equally

**Corresponding Author:**

Alona Courtney MBChB BSc(Hons) MSc MRCS

HCA Healthcare UK, 2 Cavendish Square, London W1G 0PU

ORCID ID: 0000-0002-0077-1994

alona.courtney@icloud.com

**Supplementary Materials - Index**

| **Supplementary Methods** |  |
| --- | --- |
| Survey questions | *pag. 3* |
| **Supplementary Results** |  |
| Data missingness analysis | *pag. 8* |

**Supplementary Methods**

**Survey questions**

**Demographics**

Q1 Are you filling in this survey for:

- Yourself (1)
- Your spouse or partner (2)
- Another relative or friend (3)

Q2 What is your age group?

- 18-24 (1)
- 25-34 (2)
- 35-44 (3)
- 45-54 (4)
- 55-64 (5)
- 65 or over (6)

Q3 What is your gender?

- Male (1)
- Female (2)
- Non-binary / third gender (3)
- Prefer not to say (4)
- Define another way (please specify) (5) __________________________________________________

Q4 What is your ethnic origin?

- White (British) (1)
- White (Other) (2)
- Black/African/Caribbean (3)
- Asian (4)
- Chinese (7)
- Mixed/Multiple ethnic groups (5)
- Other (6)

Q6 What is your relationship status?

- Married (1)
- Widowed (2)
- Divorced (3)
- Separated (4)
- Single (5)

Q7 What is your current employment status?

- Full-time employment (1)
- Part-time employment (2)
- Unemployed (3)
- Self-employed (4)
- Student (5)
- Retired (6)

Q13 Do you consider yourself to have a disability?

- Yes, I have a disability (1)
- No, I do not have a disability (2)
- Prefer not to say (3)

**Breast abscess history**

Q5 Have you had a breast abscess before?

- No (1)
- Yes (2)

Q8 Was it the first time or have you had a breast abscess before?

- First time (1)
- Had it once before (2)
- Had it more than once before (3)

Q9 Were you admitted to hospital for treatment?

- No (3)
- Yes - for 1 night (4)
- Yes - for 2-3 nights (5)
- Yes - for more than 3 nights (6)

Q10 Were you given antibiotics via a drip into your vein?

- Yes - just a one-off dose (1)
- Yes - multiple doses (4)
- No (2)
- Not sure (3)

Q12 Did you have drainage of pus using a needle?

- Yes, just once (1)
- Yes, two or three times (2)
- Yes, more than three times (3)
- No (4)
- Not sure (5)

**Breastfeeding**

Q14 Were you breastfeeding at the time?

- Yes (1)
- No (2)
- Not sure (3)

Q15 Were you given breast feeding advice?

- Yes (1)
- No (2)
- Not sure (3)

Q16 Did you have to pause breast-feeding from the affected breast?

- Yes, and I did not restart (1)
- Yes, but I was able to restart later (2)
- No, I carried on breast feeding (3)
- Not sure (4)

Q17 Has it impacted your ability to breastfeed long-term?

- Yes (1)
- No (2)
- Not sure (3)

Q18 Has it affected your confidence to breastfeed in the future?

- Yes (1)
- Maybe (2)
- No (3)
- Not sure (4)

Q25 Did you have to separate from your baby to undergo treatment? (Select all that apply)

- Yes, because I was admitted to hospital (1)
- Yes, because I underwent surgery (2)
- No (3)
- Not sure (4)

Q32 My treatment **negatively** affected my ability to bond with my baby

- Strongly disagree (1)
- Disagree (2)
- Neither agree nor disagree (3)
- Agree (4)
- Strongly agree (5)

Q33 Has it affected your desire to breastfeed in the future?

- Yes (1)
- Maybe (2)
- No (3)
- Not sure (4)

**Surgical history**

Q11 Did you have surgery which involved a cut to your breast in order to drain the pus?

- Yes (1)
- No (2)
- Not sure (3)

Q19 How long did it take for the wound to heal?

- Less than 2 weeks (1)
- 2-4 weeks (2)
- 4-8 weeks (3)
- More than 8 weeks (4)

Q20 How many dressing changes did you require?

- Less than 5 (1)
- 5 - 10 (2)
- More than 10 (3)

Q21 How many further hospital visits did you require?

- 0 (4)
- 1-2 (1)
- 2-5 (2)
- More than 5 (3)

Q22 Has the surgery affected the appearance of your breast?

- Yes (please specify) (1) __________________________________________________
- No (2)
- Not sure (3)

Q23 Has the surgery resulted in any scarring?

- Yes (please specify) (1) __________________________________________________
- No (2)
- Not sure (3)

**Impact on Quality of Life**

Q24 Has the treatment had any other negative repercussions in your life?

- Yes (please specify) (1) __________________________________________________
- No (2)
- Not sure (3)

Q26 How satisfied are you with the overall outcome of the treatment you received for your breast abscess?

- Extremely dissatisfied (1)
- Somewhat dissatisfied (2)
- Neither satisfied nor dissatisfied (3)
- Somewhat satisfied (4)
- Extremely satisfied (5)

Q27 How satisfied are you with your breasts now compared to before you had your breast abscess?

- Extremely dissatisfied (1)
- Somewhat dissatisfied (2)
- No change (3)
- Somewhat satisfied (4)
- Extremely satisfied (5)

Q28 My breast abscess treatment has left me **negatively** affected in the following areas: Mental health / well-being

- Strongly disagree (1)
- Disagree (2)
- Neither agree nor disagree (3)
- Agree (4)
- Strongly agree (5)

Q29 My breast abscess treatment has left me **negatively** affected in the following areas: Sexual well-being

- Strongly disagree (1)
- Disagree (2)
- Neither agree nor disagree (3)
- Agree (4)
- Strongly agree (5)

Q30 My breast abscess treatment has left me **negatively** affected in the following areas: Self confidence

- Strongly disagree (1)
- Disagree (2)
- Neither agree nor disagree (3)
- Agree (4)
- Strongly agree (5)

Q31 My breast abscess treatment has left me **negatively** affected in the following areas: Physical well-being

- Strongly disagree (1)
- Disagree (2)
- Neither agree nor disagree (3)
- Agree (4)
- Strongly agree (5)

**Supplementary results**

# Data missingness analysis

There were 320 (6.90%) missing values in the final data set of 4636 values. Cohort 4 had the highest frequency of missing values of 16.02% (Table S1).

Little’s test of missingness was performed for Cohort 1, 2 and 4 to determine the significance of missing values because these cohorts were missing between 5% and 40% of data. Little’s test suggested significance of missing values for Cohort 1 and 4. It was assumed that data were missing at random and multiple imputation was performed.

Comparison of imputed and complete cases using Chi-square test did not identify any significant differences (Table S1).

| Table S1. Number and frequencies of missing values in each of the variable in each cohort. | | | | | | | | | |
| --- | --- | --- | --- | --- | --- | --- | --- | --- | --- |
|  | **Cohort 1** | | **Cohort 2** | | **Cohort 3** | | **Cohort 4** | | **Imputed vs complete (p-value)** |
|  | **n** | **%** | **n** | **%** | **n** | **%** | **n** | **%** |  |
| **Q1** | 0 | 0 | 0 | 0 | 0 | 0 | 0 | 0 | 0.753 |
| **Q2** | 0 | 0 | 1 | 1.1 | 0 | 0 | 0 | 0 | 0.685 |
| **Q3** | 1 | 3.3 | 0 | 0 | 0 | 0 | 0 | 0 | n/a |
| **Q4** | 0 | 0 | 0 | 0 | 0 | 0 | 0 | 0 | 0.833 |
| **Q5** | 0 | 0 | 0 | 0 | 0 | 0 | 0 | 0 | N/a |
| **Q6** | 0 | 0 | 0 | 0 | 0 | 0 | 0 | 0 | 0.980 |
| **Q7** | 0 | 0 | 0 | 0 | 0 | 0 | 1 | 2.3% | 0.881 |
| **Q8** | 0 | 0 | 0 | 0 | 0 | 0 | 0 | 0 | 0.863 |
| **Q9** | 6 | 20 | 13 | 14.9 | 0 | 0 | 0 | 0 | 0.947 |
| **Q10** | 6 | 20 | 13 | 14.9 | 0 | 0 | 0 | 0 | 0.342 |
| **Q11** | 0 | 0 | 2 | 2.3 | 0 | 0 | 0 | 0 | 0.790 |
| **Q12** | 6 | 20 | 13 | 14.9 | 0 | 0 | 0 | 0 | 0.845 |
| **Q13** | 0 | 0 | 0 | 0 | 0 | 0 | 0 | 0 | 0.901 |
| **Q14** | 0 | 0 | 0 | 0 | 0 | 0 | 0 | 0 | 0.635 |
| **Q15** | 1 | 3.3 | 0 | 0 | N/a | N/a | N/a | N/a | 0.570 |
| **Q16** | 0 | 0 | 0 | 0 | N/a | N/a | N/a | N/a | 0.982 |
| **Q17** | 0 | 0 | 0 | 0 | N/a | N/a | N/a | N/a | 0.922 |
| **Q18** | 1 | 3.3 | 1 | 1.1 | N/a | N/a | N/a | N/a | 0.681 |
| **Q19** | 2 | 6.7 | N/a | N/a | 0 | 0 | N/a | N/a | 0.879 |
| **Q20** | 3 | 10 | N/a | N/a | 0 | 0 | N/a | N/a | 0.094 |
| **Q21** | 2 | 6.7 | N/a | N/a | 0 | 0 | N/a | N/a | 0.891 |
| **Q22** | 2 | 6.7 | N/a | N/a | 0 | 0 | N/a | N/a | 0.915 |
| **Q23** | 2 | 6.7 | N/a | N/a | 0 | 0 | N/a | N/a | 0.489 |
| **Q24** | 4 | 13.3 | 5 | 5.7 | 0 | 0 | 21 | 47.7% | 0.800 |
| **Q25** | 14 | 46.7 | 0 | 0 | N/a | N/a | N/a | N/a | 0.760 |
| **Q26** | 4 | 13.3 | 4 | 4.6 | 0 | 0 | 21 | 47.7% | 0.758 |
| **Q27** | 4 | 13.3 | 6 | 6.9 | 0 | 0 | 21 | 47.7% | 0.173 |
| **Q28** | 4 | 13.3 | 5 | 5.7 | 0 | 0 | 21 | 47.7% | 0.541 |
| **Q29** | 6 | 20 | 9 | 10.3 | 1 | 9.1 | 21 | 47.7% | 0.435 |
| **Q30** | 7 | 23.3 | 8 | 9.2 | 1 | 9.1 | 21 | 47.7% | 0.926 |
| **Q31** | 6 | 20 | 7 | 8 | 1 | 9.1 | 21 | 47.7% | 0.621 |
| **Q32** | 0 | 0 | 0 | 0 | N/a | N/a | N/a | N/a | 0.943 |
| **Q33** | 0 | 0 | 1 | 1.1 | N/a | N/a | N/a | N/a | 0.826 |
| **TOTAL** | 81 | 8.18 | 88 | 3.61 | 3 | 1.05 | 148 | 16.02 |  |
| **Number of values** | 990 | | 2436 | | 286 | | 924 | |  |
| **Number of participants** | 30 | | 87 | | 11 | | 44 | |  |
| **Little’s test (p-value)** | <0.001 | | 0.885 | | N/a | | 0.002 | |  |
| **Type of missing data** | Assumed MAR | | MCAR | | Negligible missing data | | Assumed MAR | |  |
